# Scaling through the pandemic: An analysis of international students’ experiences

**DOI:** 10.1177/14782103231192338

**Published:** 2023-07-26

**Authors:** Suvi Jokila, Kalypso Filippou

**Affiliations:** Department of Education, University of Turku, Turku, Finland; Department of Teacher Education, 8058 University of Turku, Turku, Finland

**Keywords:** Scale, scaling, international student, experience, Finland, university

## Abstract

In educational research, scales are often presented as the ontological reality of a study. This study problematises this starting point and suggests approaching scales as experienced and epistemically used. Thus, this study asks how international students studying at Finnish universities during the early phase of the COVID-19 pandemic negotiated their experiences using scales and scaling. The analysis was based on the responses (*n* = 84) to an open-ended question in an online survey. The survey was distributed in the spring of 2020 to international students studying in universities across Finland. We conducted an abductive analysis of how the international students employed scales in their reflections on their lives during the pandemic. Theoretically, we draw from the conceptualisation of scales, which we suggest provides an analytical lens for understanding subjective experiences, and rescaling, which students used in making sense of their experiences. We argue that international students made sense of their everyday lives during the pandemic through scales and rescaling, contributing to their sense of belonging and sense of (in)equality.

## Introduction

A wide body of research has aimed to understand international students’ experiences, especially in their host countries. This can be connected to a significant global increase in mobile student numbers, from 3.8 million in 2010 to 6.3 million in 2020 (UIS, 2023). This has resulted in the international student body becoming more heterogeneous and, along the way, expanding, for instance, the proportion of self-paying students. In a globally interconnected world, where international students are perceived as a voluntarily relocating group with positive policy objectives, the management of different spheres of their transnational lives is sometimes taken for granted. The study-abroad experience includes not only elements of mobile life, such as being part of new social contexts and academic environments, but also financial issues related to scholarships, cost-of-living expenses, and work. In fact, the international student mobility literature often approaches students’ experiences through national case studies or comparisons of the case studies, mostly in their host destinations (e.g. Darmody et al., 2022; Guo & Guo, 2017; Perez-Encinas & Rodriguez-Pomeda, 2019). Despite students originating from different contexts, their destination is often perceived as the scale of analysis rather than analysing how scales are used and experienced by the students in their meaning-making practices. Few studies focus on scales as lived experience (e.g. Prezeres, 2018; Spangler, 2022). We contribute to this body of literature by identifying scalar negotiations, which we refer to as *rescaling*, used by international students to understand their pandemic experiences. Understanding scales as experienced and lived can expand our knowledge of how individuals, such as international students, are connected to them and how the scale as a category has meaning beyond an analytical tool.

Drawing from Papanastasiou’s (2017) and Fraser’s (2010) conceptualisation of scales and combining it with Masuda and Crooks (2007) on scales as a lived experience, this study examines international students’ reflections during the early stage of the pandemic. The data are based on international students’ early pandemic responses (*n* = 84), which were expressed in a rapid-response survey in May 2020 in Finland. The time period of the pandemic provides an interesting departure for this study, as the scales became visible in an unforeseen way within the context of international students’ mobility. After years of steady increase and policy enthusiasm for international students’ mobility in higher education, the COVID-19 pandemic brought the sharpest decline in student flows ever seen, while disrupting ongoing study-abroad experiences. While the pandemic brought into the limelight the transnational character embedded in the everyday lives of mobile students, the borders crossed (and not crossed) became equally visible for study-related travel and the significance of national policies guiding citizens.

This study draws on the experiences of international students studying at universities in Finland during the outbreak of the pandemic. Finland was one of the countries that experienced relatively minor impacts from the pandemic. The pandemic measures were centralised and led by the government, with the help of health experts and authorities (Moisio, 2020). Similar to many other countries, mobility, both internationally and intranationally, was restricted, which then restricted the lives of mobile people. In addition, universities followed recommendations and moved their teaching and activities online from the end of March 2020 onwards. This pandemic framework – mostly including recommendations rather than strict obligatory measures (Moisio, 2020) – formed a new socio-economic and cultural framework for international students to navigate their lives. Similar to many other contexts, in Finland, temporary work was often lost, resulting in some international students also losing the income that they needed to maintain their financial stability. The Finnish government alleviated higher education students’ situations by lowering the credit threshold used to allocate student support (TS, 2020). However, these measures did not include international students, as they did not have direct financial support from the government.

We begin by introducing the field from which we draw our case study: international students’ mobility policies throughout the world and in Finland. Subsequently, we continue with an introduction to scales in education research, followed by a discussion of our empirical approach with the analysis and, finally, discussion.

## International students’ mobility policies throughout the world and in Finland

One of the main trends in global education has been the steady increase in students moving abroad to pursue their studies. UNESCO has defined *internationally mobile students* as follows:

… individuals who have physically crossed an international border between two countries with the objective to participate in educational activities in the country of destination, where the country of destination of a given student is different from their country of origin. … Any transfer between different education systems which does not involve the physical crossing of an international border is not considered as international student mobility. (UNESCO, 2015)

*International student mobility* refers to both short-term and long-term studies abroad. For example, one of the most well-known study-abroad programmes is the ERASMUS+ exchange programme, which can last between 3 and 12 months. Its participants attend courses and seminars, perform laboratory work, and prepare or conduct research for a thesis. Another type of international student mobility is when students complete an entire degree in another country (at the bachelor’s, master’s, or doctoral level). Hence, this is considered a degree study-abroad mobility. For this study, we have included international students with different statuses, including exchange students and students pursuing bachelor’s, master’s, and doctoral degrees. Although they form very different sets of international student groups, in their reflections, they nevertheless experienced mobile lives during the ongoing pandemic situation.

Globally, the expansion of international student mobility is evident. At the end of the 1990s, there were approximately two million international students, and this number was more than five million in 2017 (UIS, 2023). The reasons for this increase are the expansion of the middle classes around the world, the lack of local educational opportunities, commercial interests, and governments’ needs for skilled immigration (Rizvi & Lingard, 2010; Ziguras & McBurnie, 2015). Following global trends, the Finnish government has set significant policy objectives regarding internationalisation and international student mobility since the late 1980s (Lehikoinen, 2006). The internationalisation strategies applied by the Ministry of Education (1987, 2001, 2009, 2012, 2017) have led to the provision of courses and entire degrees taught in English. These efforts made Finland one of the leading providers of English-taught programmes in Europe (Wächter & Maiworm, 2014) and increased the number of international degree students from 6877 in 2001 to 20,868 in 2020 (Finnish National Agency for Education, 2018, Finnish National Agency for Education, 2022a). Despite the introduction of tuition fees for non-EU/EEA students in 2017, almost half of the student cohort usually comes from Asia. More specifically, in 2020, almost 9700 students came from Asia, about 7800 from Europe, over 2000 from Africa, over 1200 from America, and 84 from Oceania (Finnish National Agency for Education, 2022b). In 2019, over 10,000 short-term exchange students visited Finland for an international mobility period of 3 months or more (Finnish National Agency for Education, 2022c).

Alongside policy objectives, the regulatory frameworks enabling students to enter and stay in the country have recently been reformed in Finland. These reforms include the provision of proof that students can afford to live in Finland for 1 year, the granting of residence permits for the duration of the entire study, and eligibility for international students to stay for 2 years after they graduate to seek work (Migri, 2022). Earlier studies have noted that the regulations shape international student experiences and produce low-paid student workers (Maury, 2020), even though these reforms include the extension of working hours from 25 to 30 h per week for students (Migri, 2022).

## Scales in educational research

There are many approaches to international student mobility studied in the prevailing literature. International student mobility studies have an embedded geographical lens (Beech, 2019), as studies predominantly refer to physical movement in different sociocultural/national contexts. Many of them approach the phenomenon through national case studies or comparisons of case studies (e.g. Darmody et al., 2022; Guo & Guo, 2017). This body of work can be criticised for its methodological nationalism (Glick Schiller, 2010), although the state context is indeed a significant definer of the student experience (Lansing & Farnum, 2017). States regulate borders, often subsidise universities and their international activities, and even articulate the national cultural brand used in student recruitment (Jokila, 2019).

Besides national case studies, another body of literature approaches international student mobility through a transnational lens, which then analyses students’ experiences in a multiscale context (e.g. Hu et al., 2022; Martin & Rizvi, 2014). For instance, Martin and Rizvi (2014) show how international students are connected through technological devices to their family and friends back home. This creates a simultaneity of being here and there in mundane living. According to Erdal (2020), researchers separate transnational and local-level (integration to a particular locality) and then it reduces to some extend into a zero-sum game, while she suggests that the various levels should be analysed simultaneously, and their connectivity theorised. Similarly, Cox (1998) highlights that networks connect scales. This contingency of scales is focal in mobility studies and, as argued here, in international students’ experiences.

Studies on scales pinpoint hierarchical structures attached to scales. Masuda and Crooks (2007) challenge these hierarchies in defining an experiential approach to scales: ‘(It) can help to move us away from rigid hierarchies towards a use of scale as a representation of micro to macro level phenomena that are salient to people’s everyday lives such as the body, home, school, community, and nation’ (p. 257). Indeed, scales (local, national, and global) are not merely ontological realities but, rather, are epistemological tools to make sense of lives and experiences, as ‘human experiences of everyday life trace the contours of a much more complex picture of social, cultural, political and economic interconnections across scales’ (Masuda & Crooks, 2007: p. 257). With support from other researchers (namely, Jonas; Kaiser and Nikiforova, and Moore), Papanastasiou (2017) finds that ‘scale categories are fundamental to the way social actors make sense of their worlds then a rejection of scale would involve a key element of social practice being left unexamined’ (p. 1062). Indeed, we aim to highlight that the policies attached to scales can become lived experiences and that their meanings may move and differentiate into a variety of experiences. Furthermore, even though scales are interconnected in spaces, they also separate experiences.

As noted here, scales are not merely ontological realities but rather facilitate knowing and understanding of the world (Masuda & Crooks, 2007; Papanastasiou, 2017). These experiences may produce feelings of belonging and non-belonging, as mobility produces situations where mobile persons, in this case international students, do not have similar citizenship rights in their local destinations (Yuval-Davis, 2007) despite the rhetoric often emphasising equality, for instance, among higher education students. In practice, some unexpected events, such as pandemics, calling for state policies may create a sense of inequality that has an affective repercussion, such as anger and despair (Bottero, 2020).

## Empirical approach

This study is part of the International Students in Times of Crisis research project. In our exploratory study, we seek to explore how international students make sense of their pandemic experiences by employing scales. Empirically, we ask: (1) What are the scales used by students? and (2) How do they use these scales in their reflections?

In May 2020, we conducted a rapid-response survey for international students studying for different degrees (exchange students, bachelor’s degree students, master’s degree students, and doctoral researchers). Due to the timing of the survey, the responses illuminated the early experiences of international students. There were 192 respondents to our questionnaire, and most respondents were master’s degree students (*n = 99*), followed by exchange students (*n* = 61), doctoral researchers (*n* = 17), and bachelor’s degree students (*n* = 15).

The responses to the open-ended questions varied from a one-sentence response to extensive and deep insights into students’ thoughts and experiences (*n* = 87). After removing three responses stating that they had nothing to share, we included 84 responses. The shortest response was 7 words, and the longest response was 595 words. In total, there were 7708 words, which equates to an average of 92 words per response. Many of the open-ended questions’ responses were lengthy and personal, which indicates that the survey and these questions in particular acted as a reflection space in which the respondents felt they could be heard during the pandemic.

The analysis is based on the responses to the open-ended question, ‘Is there something you would like to share related to your life situation since the COVID-19 pandemic?’ We included this open-ended question in our survey to encourage our respondents to openly share information and details about their experiences during this unique period (see Allen, 2017). The respondents could answer this question without length or time restrictions to allow for the development of more mindful reflections. Furthermore, we wanted to let our participants write unexpected responses and provide them with ‘a greater *sense of engagement*’ (Singer & Couper, 2017: p. 128) while we were also seeking ‘more richly *textured data*’ (Singer & Couper, 2017: p. 128). The open-ended survey data had limitations. As the open-ended question was set rather broadly, the subjects could choose how and what kind of content they would focus on. Yet, as the aim of this study is not to produce generalised knowledge but rather to be exploratory in the experiential notions of scale in international students’ experiences, we find this open-ended question suitable for our analysis. For Braun et al. (2021), the critique of qualitative surveys’ perceived loss of depth is ‘based on imagining what qualitative surveys cannot offer and an idealisation of what interviews will offer’ (p. 644); however, the perceived anonymity of an online survey ‘can facilitate participation and disclosure in sensitive research’ (Braun et al., 2021: p. 645).

We conducted abductive content analysis (Tavory & Timmermans, 2014), where we aimed to develop the theorisation along the way. The starting stage included several readings of the responses to investigate what and how scales were used in them. During the data analysis, we used NVivo software. Alongside the data readings and analysis, we built a theoretical and conceptual understanding of the scales’ usages. First, we identified and coded five scales (transnational, state, city/town, university, and home, i.e. the house in which one stays) in the students’ reflections (see Table 1). Home scale refers to a house in which one resides (not, for instance, home as a notion of home country/parental home, if that is different to where one resides) but also a space that can facilitate, for instance, social relations. University refers to references to the institutions, for instance, as an actor in exchange students’ decisions to stay or leave. City/town denotes any such place named in the reflections. The state referred to Finland, often focussing on any state-related policies, such as immigration. The transnational scale reflected experiences in relation to countries, places, or social relationships beyond Finland. Even though it can be argued that the transnational scale is an embedded scale in international students’ lives, in our analysis, we separated it out, as not all our respondents referred to it in their reflection.

**Table 1.** Examples of codified data.

	Home	University	City/town	State (Finland)	Transnational
Extract example	It was a good thing at the beginning to be alone with myself. But with time, I began to feel alone [and] to waste my time	Overall, though, I have felt supported by the school. It has been extremely generous and helpful that degree exams could be pushed forward and study plans extended into the autumn	I would not have stayed in (name of the city) if I hadn’t had the chance to meet the people I mentioned above	There is less and less money in my bank account I am not sure that I will be able to get a residence permit for next year, as I don't have money	I'm from the US. Everything is falling apart over there, so I'm glad I'm completely safe from that, but all of my friends and family over there are stuck in the mess that is the US, as it is handling everything as poorly as possible

We examined the identified scales and analysed how they were used in students’ reflections. We then found that the scales had an organic dimension in that they were used to rescale experiences. This was due to rescaling policies that, for instance, recommended that students stay at home.

## Scaling through everyday experiences

The pandemic policy of social distancing produced a situation in which most responding international students needed to reimagine their current lives due to pandemic policies that shaped their social and financial situations. In reading the reflections of the international students on an open-ended question, most students reflected on their experiences through scales, namely the home (as a place to live), the university, the town/city level, the state, and transnational. In the usage of scales, we could observe how international students employed and needed to change the scale—the process we term *rescaling*. We identified two rescalings: downscaling and interscaling.

## Downscaling

Due to university closures and recommendations to avoid social gatherings, life centred on homes, with limited social contact or possibilities for differentiating diverse spheres of life. For example, in the following extracts, the respondents reflect on the downscaling of their lives spatially into the home space as a kind of micro-unit where all activities took place:

*Usually, I have no problems working or being at home. But I think, due to the long emergency situation and closure of institutions, it has become a bit boring and monotonous at home.* (Participant 126, doctoral researcher)

*The hardest part is to separate the home from the work environment when one is forced to work from home.* (Participant 137, master’s degree student)

Temporality is evident here, as already in May 2020, not more than 2 months after the university closures and other pandemic measures being implemented, the effect on life is perceived as significant. Home is not only a place but also a social space (Massey, 1994). Despite having the possibility to communicate online, there was a desire to meet face-to-face as students started to feel lonely. While some felt the distress of rescaling to home spaces, for others, this was not a new issue – a phenomenon observed in other studies related to international students (Sawir et al., 2008). For some respondents, staying at home meant a realisation of their pre-pandemic lonely life in Finland, which was not considered much different from their life during the pandemic:

*I am very lonely all the time, so my social life didn’t change so much. Most people are complaining now about their current social life, which is my usual social life. That made me realise even more how poor my social life was.* (Participant 85, master’s degree student)

Students who did not find their home space uncomfortable highlighted the social connections they had either at home (mostly a spouse) or at close proximity with the possibility of meeting regularly:

*Living with my partner has helped mitigate the psychological effects of social distancing. I don’t feel isolated, and I think it would have been much more difficult if I were living alone.* (Participant 13, master’s degree student)

This shows how social connections facilitated (or not) downscaling. Participant 13’s reflection highlights the home place as a space that encompasses social relations. These reflections also point out physical socialising, as the pandemic did not prevent people from meeting online.

## Interscaling

One significant, and arguably distinctive, characteristic identified in the international students’ reflections was the co-existence of different scales and, moreover, the interconnectivity by which the reflections coupled scales. This is most evident in the experiences at the state scale, for instance, losing a job due to pandemic policies, and the transnational scale, that we see, for example, in leaving no other option than to return home. This kind of connectivity of different levels is referred to as multi-layered citizenship by Yuval-Davis (2007, p. 562) to highlight that ‘people are citizens simultaneously in more than one political community’. For example, one of the master’s students illustrated the connectivity of economic instability produced by pandemic policies within the state and its relation to being able to stay in Finland. The interconnectivity here has a domino effect:

1) I am from (home country), and we are in economic sanctions. We don’t have any bank connection with other countries. My parents cannot send money to Finland for me.

2) I worked during my education, but now I am unemployed.

3) I applied for ‘unemployment benefits’, but very, very, very hard (to do) because all the websites are in Finnish and Swedish.

4) My request for unemployment benefits was rejected.

5) *Now, I am forced to sub-rent my room and go to* (home country). *Going to* (home country) *is an extra expense for me.*

(Participant 19, master’s degree student)

This reflection shows how losing a job and having limited access to information and, finally, actual economic benefits without parental support positions international students outside of the Finnish state. Many international students living in Finland, with its rather high living costs, rely on temporary work (Maury, 2020; Saari et al., 2020). In this kind of crisis situation, international students are not included in state policies, and citizenship suddenly becomes a key issue. Furthermore, the extract highlights the interconnectivity of scales in such a way that in the analysis, we cannot separate the scales but, rather, they need to be studied simultaneously.

During the pandemic period, the Finnish government was acting to support Finnish students, who were understandably troubled by the situation. Yet, similar measures were not taken with international students, as they do not hold the citizenship status that would enable them to receive government support for their studies. This produced a sense of non-belonging to Finland on a state scale and unmet expectations of how the welfare state should act in a situation such as the pandemic. Participant 163’s reflection highlights the complex meanings of home and what it would mean to return to their home country (Pérez Murcia & Boccagni, 2022):

*I don’t want to go home. I consider Finland my home for the time being, and I highly appreciate the level of education, which is why I sacrificed my amazing job and comfort in the first place to come here. But I had higher expectations that during this difficult time, the welfare state would do more for the intellectuals it educates.* (Participant 163, master’s degree student)

The state is a space where policies and social relations facilitate the sense of belonging. In the pandemic period, state policies created othering experiences and exclusion from the state benefits that had been marketed to them (Jokila, 2019). Exclusion from the state creates a sense of non-belonging to the nation as well (Yuval-Davis, 2007):

*The illusion of ‘community’ broke—I no longer believe that I am equal to other people here who have the same kind of problems as me, but have a Finnish spouse, passport or connections.* (Participant 103, master’s degree student)

This kind of loss of a sense of belonging speaks to a wider acknowledgement of communities of belonging. McMillan and Chavis (1986) define ‘a sense of community as a feeling that members have of belonging, a feeling that members matter to one another and to the group, and a shared faith that members’ needs will be met through commitment to be together’ (p. 9). Hence, international students imagine and expect that the community in their host country will work for them.

In Participant 163’s reflection, we can also see how the pandemic created a situation where some students not only constructed a sense of their own situation but also a sense of inequality (Bottero, 2020) in relation to others within Finland and even globally. This is produced in a situation where students seemingly occupying the same scale (e.g. the university or state scale) were not treated in an equal manner. This sense of inequality creates an affective situation that is ‘more a question of the embodied dispositions and emotions produced by subordination, in self-restricting choices and feelings of shame, resignation, and despair’ but also ‘dissent, anger, indignation, and struggles for greater recognition, respect and dignity’ (Bottero, 2020: p. 89):

*I understand that my situation has caught everyone off guard, but international students seem to be the pit of the problem, since we are neither Finnish nor refugees nor emigrants. To make my situation even worse, I’m not even an EU citizen. I have never felt so strongly that studying internationally is a privilege, and it’s a rocky road to make it internationally.* (Participant 163, master’s degree student)

This reflection supports an understanding that international students are perceived as a privileged group that can enjoy their mobile lives without support. However, as pointed out by many previous authors (e.g. Waters & Brooks, 2021), the international student body has become more heterogeneous.

Students’ responses can also be perceived as a mirror to the marketing of Finland and Finnish higher education, which portray Finland as a welfare state (Jokila, 2019). In such a crisis condition, subjective expectations remained unmet. In addition, global inequalities were evident in the reflections that contrasted their position in Finnish society with that of Finnish citizens. This kind of social ordering (Massey, 1994), created by the different opportunities on different scales, was lived by some of our subjects. Some reflections demonstrate that international students are here understood as visitors, and support structures are rescaled to their sending/home countries.

Living transnational lives is now enabled by technological development. This can take the form of performing rather mundane activities with one’s family in another country (Martin & Rizvi, 2014). The pandemic brought disconnectedness to experiencing these scales, as some students reported worrying about their families at home, even causing a sense of not knowing what was happening in their local spaces in Finland. The situation in Finland was not as severe as in many other parts of the world, creating uneven circumstances of worry and mental distress:

*It was a strange situation because my body was here, but my mind and all my energy were following the news in [home country] and in [home area], talking with my family and my friends, who were there. So, I felt disconnected about the Finnish situation. I asked some teachers how the situation was here to know their point of view, but I was spending too much time reading news from my home country—more than from here. So I’m completely disconnected about what is happening here. When will the museums open again, the swimming pools, the libraries … how is the mobility in (city) area … I have no idea about all these questions.* (Participant 95, exchange student)

Participant 95’s reflection illustrates the loss of sense of the local situation, even in the city space they are living in, due to worries over family. Besides the very immediate rescaling of everyday living, future imaginaries were also at stake, as the pandemic created unexpected uncertainty. This was experienced in various ways, including:

*I was working here in Finland, and I want to stay to look for a doctoral position here. However, the new situation has become complicated to handle, as I’m not sure if it would be possible for me as a foreigner to find a job or a PhD position in the complex social and economic situation after the coronavirus.* (Participant 4, master’s degree student)

Indeed, what we could observe here is that the scales were also connected in future imaginaries. Respondents used a comparison of scales to point out different situations at various scales and the potential future imaginary. Comparing scales between Finland and the home country was employed in the reflection on the pandemic measures taken by the Finnish government and the wider possibilities for social distancing due to the low density of inhabitants, which was often perceived in a positive light:

*In early March, my home university sent me multiple emails requesting that I make arrangements to return home. There were several reasons why I didn’t want to go home, mainly because I felt safer in Finland and simply did not want to leave, but my home university did not accept my decision easily.* (Participant 76, exchange student)

While the Finnish government and Finnish universities did not require or recommend that international students return home, some decided to go, as their home countries or institutions were enacting such a policy. International students responding to the survey had temporal expectations of their stay and were particularly sad about it being shortened. Personal experiences and governmental regulations sharply contrasted at times. International students received recommendations from (or were even forced by) their home universities and governments to return, while the Finnish government did not implement such policies.

## Conclusion

We have presented an analysis of how international students studying in Finnish universities at the beginning of the COVID-19 pandemic negotiated their lived experiences (Masud & Crooks, 2007) and employed scales in doing so. We examined scales with an experiential approach, allowing us to understand scales – often presented as ontological realities – as epistemology (Papanastasiou, 2017) and experienced (Masuda & Crooks, 2007). We follow Jones (2017) and Paul and Yeoh (2020) in arguing against essentialism, which refers to a tendency not to perceive a particular migration group’s heterogeneity but rather to call for nuanced and complex ways to experience scales in everyday lives (Masuda & Crooks, 2007).

We have identified transnational, state, city/town, university, and home scales in international students’ reflections that shaped their everyday lives and future imaginaries. These identified scales include downscaling and interscaling. Downscaling refers to the experience of staying at home and how that contributes to the emotions and social exclusion it produces for some, while others can cope with the situation due to their social relations. The analysis shows that this rescaling is not equally available to all. Not all students can sense boredom or travel if necessary in this unexpected situation; rather, they may face harsh circumstances. There is inequality in what kind of rescaling the students can do and what some are forced to do.

We also showed the interconnectivities of the scales and how they may contribute to students’ experiences in various ways. We called this *interscaling* to illuminate how scales are connected and how these connectivities may contribute to a sense of non-belonging (Yuval-Davis, 2007) and a sense of inequality (Bottero, 2020). Hence, this contributes to educational policy studies by using scales as an analytical lens to show how higher education, migration, and pandemic policies in Finland and transnationally are intertwined in the subjective experiences of international students. This study builds on existing studies on scales in understanding international students’ experiences on different scales: university (Cena et al., 2021), home (Spangler, 2022), state (Lansing & Farnum, 2017), city (Prezeres, 2018), and transnational (Martin & Rizvi, 2014) scales, and highlights the multiscalarness and interconnectivity of the scales. For instance, an experience of employment may have resulted in financial hardship or uncertain future imaginaries showing the connectedness of the many scales that international students are living in. Thus, the policies implemented during the pandemic differentiated international students from local students (e.g. provided subsidies) and also differentiated the international student body (e.g. the income level of the students’ home country).

A central issue during the pandemic in Finland was state policies to mitigate the virus, which then meant that the state stretched other scales in an unforeseen manner. While the transnational scale was ‘open’ to mobility pre-pandemic, during the pandemic, students needed to negotiate and consider whether to return home. This negotiation was attached to the national conditions in Finland, with some hoping to stay and others worrying that the financial situation would not allow them the opportunity to stay in Finland due to lost jobs and so on.

This study is exploratory in that we employed students’ reflections in a broadly defined open-ended question. While we did not limit or guide their reflections, we could not narrow their focus and ask further questions, which can be perceived as a limitation of the study. This is also a benefit in that students can freely reflect on their experiences in the open-ended question provided. The aim of this analysis was not to provide generalisable knowledge but rather to explore the experiences of scales and their usage in individual reflections and, hence, to show that scales in educational research need further understanding in the everyday usage of students and other actors.

This article has shown that in order to understand international students’ experiences comprehensively, we need to move from perceiving scales as the ontological backbone of the study, often located in one country context, to identifying the variety of scales shaping international students’ lives and how they contribute to international students’ experiences. Thus, this study has yielded insights in moving to theorise scales as present in everyday practices and reflections (Masuda & Crooks, 2007).
